# The changing landscape of nuclear medicine and a new era: the “NEW (Nu) CLEAR Medicine”: a framework for the future

**DOI:** 10.3389/fnume.2023.1213714

**Published:** 2023-06-27

**Authors:** Mehdi Djekidel

**Affiliations:** Radiology/Nuclear Medicine, Northwell Health, New York, NY, United States

**Keywords:** nuclear medicine, PET, SPECT, artificial intelligence (AI), theranostics, software, hardware—emerging technologies—emerging interfaces

## Abstract

Nuclear Medicine is witnessing a revolution across a large spectrum of patient care applications, hardware, software and novel radiopharmaceuticals. We propose to offer a framework of the nuclear medicine practice of the future that incorporates multiple novelties and coined as the NEW (nu) Clear medicine. All these new developments offer a significant clarity and real clinical impact, and we need a concerted effort from all stakeholders in the field for bedside implementation and success.

The expansion of nuclear medicine within the clinical arena over the past 2 decades has become quite “CLEAR”. This truth has become self-evident over the past 10 years to all stakeholders and non-stakeholders through the expansion of fluorodeoxyglucose positron emission tomography (FDG PET) for oncology indications through the National Oncologic PET Registry (NOPR) as well as recent removal of centers for Medicare & Medicaid services (CMS) barriers and other insurance providers for non-oncology indications (1). Recent Food and Drug Administration (FDA) and European Medicines Agency (EMA) approval of several personalized, targeted radiopharmaceuticals for diagnosis and therapy brings more clarity to clinical management Tables 1, 2 (2–18). Novel clinical trials such as the Imaging Dementia-Evidence for Amyloid Scanning (IDEAS) Study and New IDEAS: Imaging Dementia-Evidence for Amyloid Scanning Study are setting a path towards clinical acceptance of imaging biomarkers in the management of dementia patients (19, 20). Novel dementia therapeutic trials have incorporated tau and amyloid imaging biomarkers to optimize patient selection and hence outcomes (21). Clinical trials are also underway for novel diagnostic and therapeutic radiopharmaceuticals across the world involving industry and industry-academic joint ventures. Leading international societies such as the European Association of Nuclear Medicine (EANM) have laid down a framework for future high quality nuclear medicine services (22). Other visionary futuristic initiatives emanate from the Society of Nuclear Medicine and Molecular Imaging (SNMMI) which is leading a novel Radiopharmaceutical Therapy Registry “RaPTR” that is set to enhance the standard, value, quality, access, and outcomes of nuclear medicine therapies, dosimetry and theranostics in clinical practice (23). On the other hand, the Radiological Society of North America (RSNA) and other worldwide collaborators have set the path towards optimizing best medical practice by leading the way towards standardization of quantitative biomarker imaging through the Quantitative Imaging Biomarkers Alliance® (QIBA), Japan-QIBA and the European Imaging Biomarkers Alliance (EIBALL) (24). The SNMMI's clinical trials network (CTN) is also leading the way in standardization and harmonization of quantitative and semi-quantitative nuclear medicine datapoints (25). Australian groups have also initiated the PLANET registry, the first dedicated registry for neuroendocrine cancers in Australia. It offers a gateway for patients to novel therapeutic radiopharmaceutical trials (26). Canadian investigators have similarly initiated a PSMA PET registry (PREP) (27). On the same note the NOBLE/Nobody Left Behind registry is an international registry evaluating PSMA SPECT utility in prostate cancer patients (28).

**Table 1:** Novel FDA approved diagnostic/imaging clinical radiopharmaceuticals that have been successfully translated from bench to bedside.

Radiopharmaceutical	FDA approval	Approved indication	Additional applications
^18^F-Piflufolastat (2)	2021	Imaging of prostate-specific membrane antigen (PSMA) positive lesions in men with prostate cancer	None
^68^Ga-Gozetotide (3)	2020	Imaging of prostate-specific membrane antigen (PSMA) positive lesions in men with prostate cancer	None
^64^Cu-DOTATATE (4)	2020	Localization of somatostatin receptor positive neuroendocrine tumors (NETs) in adult patients	• Meningiomas • Oncogenic Osteomalacia • Esthesioneuroblastoma • Pheocromocytoma • Medullary Thyroid Carcinomas • Small Cell Lung Cancer • Merkel Cell Carcinoma • Iodine negative differentiated thyroid cancer
^18^F-FES fluoroestradiol (5)	2020	Detection of estrogen receptor (ER)-positive lesions as an adjunct to biopsy in patients with recurrent or metastatic breast cancer	None
^18^F-flortaucipir (6)	2020	Estimate the density and distribution of aggregated tau neurofibrillary tangles (NFTs) in adult patients with cognitive impairment who are being evaluated for Alzheimer's disease (AD)	None
^18^F-DOPA (7)	2019	Evaluation of adult patients with suspected Parkinsonian syndromes	• Congenital Hyperinsulinism • Pheochromocytoma • Paraganglioma • Neural Crest Tumors • Neuroendocrine Tumors • Brain Tumors
^68^Ga-DOTATATOC (8)	2019	Localization of somatostatin receptor positive neuroendocrine tumors (NETs) in adult and pediatric patients	• Neuroblastoma • Meningiomas • Oncogenic Osteomalacia • Esthesioneuroblastoma • Pheocromocytoma • Medullary Thyroid Carcinomas • Congenital Hyperinsulinism • Small Cell Lung Cancer • Merkel Cell Carcinoma • Iodine negative differentiated Thyroid Cancer
^68^Ga-DOTATATE (9)	2016	Localization of somatostatin receptor positive neuroendocrine tumors (NETs) in adult and pediatric patients	• Neuroblastoma • Meningiomas • Oncogenic Osteomalacia • Esthesioneuroblastoma • Pheocromocytoma • Medullary Thyroid Carcinomas • Congenital Hyperinsulinism • Small Cell Lung Cancer • Merkel Cell Carcinoma • Iodine negative differentiated Thyroid Cancer
^18^F-Fluciclovine (10)	2016	Imaging in men with suspected prostate cancer recurrence based on elevated blood prostate specific antigen (PSA) levels following prior treatment	None
^18^F-florbetaben (11)	2014	Estimate β-amyloid neuritic plaque density in adult patients with cognitive impairment who are being evaluated for Alzheimer's disease (AD) or other causes of cognitive decline	None
^18^F- Flutemetamol (12)	2013	Estimate β-amyloid neuritic plaque density in adult patients with cognitive impairment who are being evaluated for Alzheimer's disease (AD) or other causes of cognitive decline	None
^18^F- Florbetapir (13)	2012	Estimate β-amyloid neuritic plaque density in adult patients with cognitive impairment who are being evaluated for Alzheimer's disease (AD) or other causes of cognitive decline	None
^11^C-Choline (14)	2012	Imaging of patients with suspected prostate cancer recurrence and non-informative bone scintigraphy, computerized tomography (CT) or magnetic resonance imaging.	None

**Table 2:** Novel FDA approved therapeutic clinical radiopharmaceuticals that have been successfully translated from bench to bedside.

Radiopharmaceutical	FDA approval	Approved treatment indication
^177^Lutetium-vipivotide tetraxetan (15)	2022	Treatment of adult patients with prostate-specific membrane antigen (PSMA)-positive metastatic castration-resistant prostate cancer (mCRPC) who have been treated with androgen receptor (AR) pathway inhibition and taxane-based chemotherapy.
^131^Iodine Iobenguane (16)	2018	Treatment of adult and pediatric patients 12 years and older with iobenguane scan positive, unresectable, locally advanced or metastatic pheochromocytoma or paraganglioma who require systemic anticancer therapy.
^177^Lutetium-DOTATATE (17)	2018	Treatment of somatostatin receptor-positive gastroenteropancreatic neuroendocrine tumors (GEP-NETs), including foregut, midgut, and hindgut neuroendocrine tumors in adults.
^223^Radium Dichloride (18)	2013	Treatment of patients with castration-resistant prostate cancer, symptomatic bone metastases and no known visceral metastatic disease.

Additionally, one should note the development of several PET probes that have made it successfully through FDA approval (2–18) and their proven clinical impact/significance. This has encompassed not only diagnostic but also therapeutic radiotracers. The main hurdles left for full rollout and explosive growth is price control, cost effectiveness and of course appropriate utilization for full patient benefit as well as a critical knowledge piece sometimes lacking within patient care teams across the world. National and international leading societies and stakeholders are setting the path for dynamic guidelines and appropriate use criteria. One should not underestimate the lack of access of appropriate tests and therapies for patients. Beyond these logistical and health economics factors an additional hurdle is this brazen diminished cutting-edge expertise across the spectrum of real-world nuclear medicine practice. More expertise is needed within the nuclear medicine community as well as the larger multidisciplinary patient care teams.

Nuclear medicine is the most rapidly growing subspecialty in radiology. The NEW CLEAR medicine is no longer fuzzy imaging, equivocal reporting, limited quantitation, limited clinical applications, poor temporal and spatial resolution, research focused field, basic science fixated, and with limited clinical translational impact. We are in a new era of precision nuclear medicine where the extent of new tools available is astounding and requires a large breadth of expertise. This is the era of the “NEW (Nu) CLEAR Medicine”.

High end nuclear medicine was historically reserved to research applications and clinical nuclear medicine was applying only basic run of the mill tools, applications, and indications. However, over the past 10 years a variety of tools have started to be incorporated as a standard of care in the nuclear medicine clinic, including wider use of hybrid imaging devices, novel hardware technology, unique crystals, materials, wider availability of indication specific radiopharmaceuticals for diagnosis and treatment. More advanced software is being incorporated into the clinic with a variety of commercial vendors modernizing their software platforms and going through the necessary FDA approval process. This encompasses independent vendors as well as scanner based pre and post processing workstations seemingly fancy but very useful and necessary.

This has completely changed clinical practice, and the new era of clarity is here “The NEW (Nu) CLEAR Medicine” or Nuclear Medicine of the Future is at our fingertips. However, what is the FRAMEWORK of this New (Nu) Clear Medicine? It embraces:

1. **New hardware technology:** An explosion of novel technology has made its way into the clinic all to the patients benefit including Total Body PET/Long Axial Field of View PET (29), PET/MRI (30); LINAC PET (31), Total Body single photon emission tomography (SPECT), CZT digital SPECT technology (32, 33), SPECT/MR as well as high end traditional SPECT/CT scanners. These allow for a personalized approach using the right tool for the right patient and to optimize radiation doses, acquisition protocols, scanning times and overall patient care for diagnosis, dosimetry, and treatment.
2. **New software technology:** Historically, mostly research software has been able to perform fully quantitative, semiquantitative or advanced subtraction techniques. Now major vendors have developed advanced software suites on scanner workstations or independent consoles, as well as online/through cloud systems. Independent commercial software is also now state of the art with advanced processing and postprocessing capabilities in SPECT and PET hybrid imaging as well as incorporating advanced techniques in cardiac, general, musculoskeletal, oncology, neuronuclear and therapy applications.
3. **Artificial intelligence (AI) technology:** The potential for artificial intelligence is huge. It can impact administered dose, scan duration, resolution, workflows as well as post processing image analysis influencing for example lesion classification, lesion characterization, and patient risk stratification with the translation of complex risk stratification nomograms into automated simplified algorithms (34, 35). AI will be of value for pre reconstruction and post processing techniques (34, 35). The aspiration of having an attenuation correction (AC) map without a CT acquisition and to analyze anatomy from prior scans is real (36). AI is also set to impact radiotracer design chemistry, labeling and the automated segmentation process as well as generating reproducible imaging biomarkers in a time saving fashion and harmonizing/standardizing expert interpretation everywhere (34, 35). AI will also be able to uncover imaging phenotypes, disease specific biological patterns, and perform image segmentation and image denoising. AI will be used in several paradigms for AI assisted care (AI as an assistant in several workflow tasks usually performed manually by an operator). AI augmented care (augmented image analysis at the pre and post processing level). AI enhancement (Image resolution enhancement). AI integrative data (integrating multimodal data elements). AI facilitated patient care such as computer aided diagnostics as well as facilitated reporting improving the expertise and human capacities of joining multiple datapoints together and increasing human capacities in visual interpretation and detection with strict Quality Control algorithms. Facilitated reporting with standardized outputs of reports based on specific algorithms and with elimination of errors will be helpful and soon an everyday reality. AI based reporting that helps incorporate image analysis data into standardized reports based on optimized information for clinical management decision making. In a more general aspect flagging within EMR and PACS systems for physicians to remember crucial protocol following, preparation and urgency of exam reporting. AI will also be critical in dosimetry protocols automation and radiomics incorporation as adjunctive tools for patient management. AI will also be essential to optimize harmonization and standardization of clinical workflows and protocols based on preset high-quality standards as well as optimizing virtual biopsies image phenotyping (37).
4. **Direct clinical patient care:** With a variety of therapies approved the new (nu) clear medicine is more patient facing than ever. More clinical background is hence needed for patient evaluations in initial treatment settings and longitudinally to optimize continuity of patient care. There is a necessity to provide more comprehensive clinical assessments pre and posttreatment beyond the radiation exposure and radiation safety paradigms with an increased importance of office visit consultations along the entire continuum of patient care as well as engaged full participation in multidisciplinary teams and building strong long term and not one time point patient-physician relationships.
5. **Dosimetry and outcomes:** Keeping in mind the heterogeneity of patient outcomes and to some extent suboptimal efficacy of radiopharmaceutical therapies. There is mounting evidence that patient specific dosimetry may guide outcomes by ensuring thresholds are being met for dose delivered to target and at-risk organs. This will encompass predictive and prognostic dosimetry paradigms applied clinically to optimize outcomes (38–41).
6. **Standardization and harmonization of tools:** Nuclear medicine practice can be quite heterogeneous across the world. There is a pressing need to follow best medical practice as well as standardization and harmonization of protocols to optimize patient outcomes. High quality is necessary not only in radiotracer production, radio pharmacy operations, acquisition protocols, but also in processing and post processing tools. This will be applied on an institutional and multi-institutional level (22).
7. **Standardized structured reporting:** Nuclear medicine reports can be ambiguous and may not offer a clear answer to the referring physician. Nuclear medicine of the future will allow for key clinical elements with proven benefit (evidence-based medicine) to be communicated clearly, consistently and with no ambiguity to patients, referring physicians and various other key stakeholders including payors. This will apply to different time points across the patient continuum of care and treatment (42, 43).
8. **Multiprobe assessments for accurate disease characterization:** Current clinical paradigm uses a single probe for diagnosis, staging or other mode of patient care. However, it is quite clear that this strategy is suboptimal and limited as it fails to fully characterize the entire phenotype of the patient's disease. Disease characterization and lesional characterization with current clinical models is quite limited. This is due to interpatient heterogeneity, intra-patient heterogeneity, inter-lesional heterogeneity as well as intralesional heterogeneity. These changes are dynamic as this heterogeneity is not static and changes dynamically with treatment. Multiparametric assessments of disease using multiple probes, advanced image analysis tools coupled to other biochemical, proteomic and genomic data in the same patient will be incorporated into routine clinical practice (44–46).
9. **Dynamic treatment modulation** is also necessary. Risk adapted treatments are needed moving away from static systems of clinical care to dynamic systems modulating patient management across the clinical patient historical continuum. Nuclear medicine offers predictive and prognostic biomarkers allowing and contributing to dynamic treatment modulation (47, 48).
10. **Biopsy guidance** through intraoperative probe utilization or through minimally invasive techniques targeting the most avid areas of a lesion or other disease relevant pathology (49).
11. **Heterogeneity and biopsy guidance** is something that needs to be addressed clinically as novel nuclear medicine techniques offer not only the opportunity for noninvasive accurate disease characterization but also can guide invasive or minimally invasive biopsy guidance towards the area of a lesion most representative of the patient's disease.
12. **Theranostics and New (Nu)clear medicine Therapeutics** are playing an increasingly primordial role clinically with several therapeutic radiopharmaceuticals approved for clinical use worldwide. The theranostic paradigm allows you to see what you treat and select the patients with the most favorable disease phenotype for best outcomes. In the future we will pay attention more closely at disease heterogeneity -best seen with functional imaging and less likely to be uncovered with traditional morphological imaging-. This will prompt combination treatments of nuclear medicine therapies and non-nuclear medicine therapies as well as the combination of two different radiopharmaceuticals targeting different disease profiles in the same patient with the same or different radionuclides. Another attractive future nuclear medicine treatment paradigm will be moving to an early first line strategy. Traditionally radiopharmaceutical therapies have been employed on end-stage heavily pretreated patients which inherently may limit its efficacy by selecting the most treatment resistant group of patients and the most vulnerable to side effects and toxicity. One example is the LuTectomy (41) trial which aims to explore the dosimetry, efficacy, and toxicity of the lutetium-PSMA treatment early on in men with high-risk localized/locoregional advanced prostate cancer with high prostate-specific membrane antigen expression who are undergoing radical prostatectomy and pelvic lymph node dissection. Other trials and novel strategies need to be investigated.

This NEW (Nu) CLEAR medicine needs to incorporate enhancements within invisible seamless paradigms. This new era needs new and more expertise and not less to ensure high quality patient care. Every patient deserves the best and it is our responsibility to ensure this “New Clear Medicine” is translated and applied clinically on a broad scale.

It is quite clear that the advancements in nuclear medicine will be in the near future applied uniformly and as standards. This undoubtedly requires strong immediate leadership and guidance from all stakeholders to train the future experts and optimize the clinical practice of current nuclear medicine experts. It is only a matter of time however Prime time is Now.

## Data Availability

The original contributions presented in the study are included in the article, further inquiries can be directed to the corresponding author.
